# Economic evaluation of digitally supported therapy for people with psychosis who hear distressing voices: the AVATAR2 trial

**DOI:** 10.1192/bjo.2025.10925

**Published:** 2025-12-15

**Authors:** Paul McCrone, Evdoxia Gkaintatzi, Thomas Ward, Clementine J. Edwards, Hassan Jafari, Richard Emsley, Mark Huckvale, Mar Rus-Calafell, Miriam Fornells-Ambrojo, Andrew Gumley, Gillian Haddock, Sandra Bucci, Hamish J. McLeod, Jeffrey McDonnell, Moya Clancy, Michael Fitzsimmons, Alice Montague, Nikos Xanidis, Hannah Ball, Thomas K. J. Craig, Philippa A. Garety

**Affiliations:** Institute for Lifecourse Development, University of Greenwich, London, UK; Department of Psychology, Institute of Psychiatry, Psychology & Neuroscience, King’s College London, London, UK; South London & Maudsley NHS Foundation Trust, London, UK; Department of Biostatistics and Health Informatics, Institute of Psychiatry, Psychology and Neuroscience, King’s College London, London, UK; Department of Speech, Hearing and Phonetic Sciences, University College London, London, UK; Mental Health Research and Treatment Center, Faculty of Psychology, Ruhr University Bochum, Bochum, Germany; Research Department of Clinical, Educational and Health Psychology, University College London, London, UK; North East London NHS Foundation Trust, London, UK; School of Health & Wellbeing, University of Glasgow, Glasgow, UK; NHS Greater Glasgow & Clyde, Glasgow, UK; Division of Psychology and Mental Health, School of Health Sciences, University of Manchester and the Manchester Academic Health Sciences Centre, Manchester, UK; Greater Manchester Mental Health NHS Foundation Trust and the Manchester Academic Health Sciences Centre, Manchester, UK; Department of Health Service and Population Research, Institute of Psychiatry, Psychology & Neuroscience, King’s College London, London, UK

**Keywords:** Cost-effectiveness, psychosis, auditory hallucinations, quality-adjusted life years, psychological support

## Abstract

**Background:**

AVATAR therapy, a digitally supported intervention, utilises avatars to promote recovery in people who experience distressing auditory hallucinations. This approach was recently evaluated in a multicentre randomised controlled trial comparing brief (AV-BRF) and extended (AV-EXT) forms of therapy with treatment as usual (TAU). There was evidence for the effectiveness of therapy, particularly for AV-EXT. However, value for money needs to be assessed.

**Aims:**

To compare separately the cost utility of the brief and extended forms of AVATAR therapy with TAU.

**Method:**

In a three-arm randomised controlled trial the use of health services was measured, and costs (2021/2022; pounds sterling) calculated from a health and social care perspective over a 28-week follow-up period. Quality-adjusted life years (QALYs; derived from the 5-level version of the EuroQol 5-Dimension questionnaire) were combined with costs.

**Results:**

AV-BRF resulted in extra costs of £319 (95% CI, −£1558 to £2496), and AV-EXT in lower costs of £1965 (95% CI, −£1912 to £1519), compared with TAU. Over the follow-up, AV-BRF resulted in 0.0159 (95% CI, −0.0103 to 0.0422) and AV-EXT in 0.0173 (95% CI, −0.0049 to 0.0395) more QALYs than TAU. The cost per QALY for AV-BRF compared with TAU was £20 016, while AV-EXT dominated TAU (lower costs and more QALYs).

**Conclusions:**

Neither version of AVATAR had a substantial impact on QALYs. However, AV-EXT did result in reduced care costs − albeit not statistically significant − and was potentially cost-effective compared with TAU. AV-BRF had an incremental cost-effectiveness ratio that indicated lower potential cost-effectiveness. These findings are uncertain, but could still inform decision-making regarding interventions in this field.

The prevalence of schizophrenia has been estimated at 0.28%, and results in impaired quality of life and high levels of disability.^1^ In addition to these impacts, schizophrenia and related conditions result in substantial economic costs associated with service use and lost work.^2,3^ Symptoms of schizophrenia and schizophrenia spectrum disorders include auditory hallucinations (hereafter ‘voices‘), and these affect up to 70% of those with the disorders.^4,5^ Voices can persist for long periods of time and cause significant distress. While therapy, either psychological or medication-based, can be effective for some individuals, many show an incomplete response.^6,7^

Interventions have been developed, and are being evaluated, that specifically address auditory hallucinations. These include an approach (Talking with Voices) that encourages understanding through dialogue with the voices,^8^ and another (Relating Therapy) that focuses on assertive responses to voices using role-play methods.^9^ Over the past decade, a digitally supported approach (AVATAR therapy) has emerged as an innovative way to help individuals experiencing distressing auditory hallucinations.^10^ It involves a therapist and voice-hearer working together to create a digital representation (‘avatar’) as a close match to the main distressing voice. The voice-hearer then engages in a series of active dialogues with their avatar, supported by the therapist, in which the aim is to reduce voice-related distress and build empowerment. The first fully powered trial (AVATAR1) compared seven sessions of AVATAR therapy against supportive counselling. AVATAR therapy showed significant reductions in the severity of voice-hearing experiences at 12 weeks post-therapy (the primary end-point), with no adverse events related to the therapy.^11^

Based on learning from the AVATAR1 trial, the first three-arm, multi-site randomised controlled trial (RCT) of AVATAR therapy has recently evaluated the brief (AV-BRF) and extended (AV-EXT) forms of AVATAR therapy, each against treatment as usual (TAU) alone.^12^ The AVATAR2 trial is the first to test effectiveness when therapy was delivered by a large cohort of therapists across geographically diverse sites. The AVATAR2 study found that both versions of AVATAR therapy significantly reduced voice-related distress at post-therapy (16-week) follow-up compared with TAU. AV-EXT therapy exceeded the prespecified threshold for a clinically significant post-treatment change on the primary outcome, while AV-BRF was slightly below this threshold. These effects were not maintained at 28 weeks, thus providing only partial support for the primary hypothesis. There were improvements in the key secondary outcomes of voice severity for both AV-BRF and AV-EXT at 16 weeks, and reduced voice frequency for AV-EXT at both time points. AVATAR therapy was found to be safe, in that there were no serious adverse events determined to be definitely related to receiving therapy.

Assessing the efficacy and effectiveness of an intervention such as AVATAR therapy is essential if it is to be recommended as a treatment for patients with distressing voices in routine care settings. However, it is also crucial to assess value for money to weigh the ‘opportunity cost’ of deployment of AVATAR therapy against alternative uses of available resource. This paper reports an economic evaluation with a primary aim of comparing the cost utility of brief and extended AVATAR therapy each against TAU in terms of quality-adjusted life years (QALYs).

## Method

### Trial design

Details of the study methods are given elsewhere.^13^ This is a three-arm, parallel-group, superiority RCT (ISRCTN Registry no. ISRCTN55682735), with a 1:1:1 allocation and blinded assessors, that has been conducted to evaluate the effectiveness of AVATAR therapy (both brief and extended versions) in individuals diagnosed with schizophrenia spectrum disorder or affective disorder with psychosis (bipolar, or depression with psychosis), when added to TAU compared with TAU alone. The trial received Health Research Authority ethical approval from London-Camberwell St Giles Research Ethics Committee (no. 20/LO/0657). All participants in the trial provided written informed consent.

### Setting and sample

Four research sites in the UK were involved in the trial: the Institute of Psychiatry, Psychology & Neuroscience (KCL), the University of Manchester, the University of Glasgow and University College London. Each of these research sites was affiliated with two local National Health Service (NHS) sites, and therapy sessions were primarily conducted in community settings. An important aim of the trial was to test effectiveness when therapy was delivered by a wider workforce, across geographically and demographically diverse locations including front-line mental health services. The study inclusion criteria were: (a) be aged 18 years or over; (b) currently under the care of a specialist mental health team (in- and out-patient settings); (c) have current frequent and distressing voices (as measured by a score of at least 1 on each of the intensity of distress and frequency items of the Psychotic Symptoms Rating Scale (PSYRATS-AH)), persisting for at least 6 months and spoken in English; (d) speak and read English to a sufficient level to provide consent and complete the assessment procedures; and (e) have a clinical diagnosis of schizophrenia spectrum disorder (ICD10 F20–29) or affective disorder with psychotic symptoms (ICD-10 F30–39, subcategories with psychotic symptoms), as determined through clinical records and additional consultation with the clinical team if unclear. Full details of the inclusion and exclusion criteria and flow through the trial are provided in the published protocol and clinical results.^12,13^

### Interventions

Participants were randomised to one of three groups: AV-EXT plus TAU, AV-BRF plus TAU or TAU alone. Both forms of AVATAR start with an initial assessment session including the avatar creation. AV-BRF then comprises six individual, face-to-face sessions delivered by trained therapists, which involve active avatar dialogues using the AVATAR therapy software where the avatar is voiced by the therapist using voice-transformation software. AV-BRF focuses on reducing anxiety through exposure to the avatar and targeting increased power, control and self-esteem. AV-EXT consists of up to 12 active dialogues and comprises 2 phases. The first phase (typically the first four to five sessions) mirrors AV-BRF. The second phase aims to build a more personalised understanding of the voice within the context of the person’s life history, and includes a wider range of potential therapy targets. Within both AV-BRF and AV-EXT, important therapeutic work also occurs pre- and post-dialogue to support positive change.

### Outcomes, service use and costs

The primary clinical outcome measure was the distress dimension of the Psychotic Symptoms Rating Scale (PSYRATS-AH).^14^ For the cost-effectiveness analysis, the combination of health and social care costs with QALYs was undertaken. QALYs were calculated based on the 5-level version of the EuroQol 5-Dimension questionnaire (EQ-5D-5L) scores at 3 specific time points: baseline, week 16 and week 28. The EQ-5D-5L tool assesses 5 domains (mobility, self-care, usual activities, pain/discomfort and anxiety/depression) on a scale from 1 (indicating no problems) to 5 (signifying extreme problems).^15^ The resulting 5-digit score represents a distinct health state, which is then converted to a value ranging from 1 (full health) to 0 (death). We used weights from the earlier EQ-5D-3L instrument utilising the eq5dmap function in Stata.^16^ Using the area under the curve method, these weights were combined at baseline and each subsequent follow-up point to generate QALYs. This approach assumes a linear change between quality-of-life scores. The costs were measured from a health and social care perspective. Service use was measured with the researcher-administered Client Service Receipt Inventory (CSRI) at baseline, week 16 and week 28.^17^ Participants were requested to indicate whether, in the period leading up to baseline or since the last assessment, they had utilised particular services including primary care, hospital-based secondary care or social care. If participants reported using these services, they were further queried about the frequency of utilisation. Regarding in-patient care, participants were asked to specify the number of days spent in hospital. Electronic clinical notes were reviewed by unblinded trial coordinators, and additional service use included in the CSRI at each time point. Costs were computed by integrating the information of therapy time received with the relevant national unit cost data for 2021/2022.^18,19^ The cost for the therapist’s time was based on a Band 8 healthcare professional, with an assumed 51% increase to account for non-contact time (based on discussions with colleagues conducting similar work). Given the probable importance of this unit cost, we conducted sensitivity analyses around it (see below). The unit costs used are available in the supplementary information available at https://doi.org/10.1192/bjo.2025.10925. The hardware and software cost depends on the number of potential users, and was estimated at £50 in the base case analysis (varied in sensitivity analyses).

### Analysis

Analysis was conducted using Stata 18.0 for Windows (Stata, College Station, Texas, USA; https://www.stata.com/) and R Studio for Windows (R Foundation for Statistical Computing, Vienna, Austria; https://www.R-project.org/). We followed recent recommendations for conducing trial-based cost-effectiveness analyses using R according to Ben et al.^20^ The main analyses were conducted with multiple imputation for missing data. Imputations were based on chained equations and predictive mean matching from the nearest five neighbours. A comparison was made between the three groups regarding the utilisation of services and their associated costs. The total healthcare costs over the follow-up period were compared between (a) the AV-EXT and TAU groups and (b) the AV-BRF and TAU groups, using seemingly unrelated regression models controlling for baseline costs and study site. QALYs were similarly compared, controlling for baseline utility. (We did not directly compare AV-BRF and AV-EXT groups.) If either arm had lower (higher) costs and produced more (fewer) QALYs than TAU, it was then considered to be ‘dominant’ (‘dominated’). If it had higher (lower) costs and produced more (fewer) QALYs, incremental cost-effectiveness ratios (ICERs) were then produced by dividing incremental costs by incremental QALYs to show the cost incurred (saved) by AV-EXT or AV-BRF to produce one more (fewer) QALY compared with TAU.

Uncertainty was addressed using cost-effectiveness planes and cost-effectiveness acceptability curves (CEACs). These were derived by repeating the multiple imputation procedure for each of 1000 bootstrapped resamples and plotting the incremental cost and QALY combinations against each other, and by generating incremental net benefits. These were defined as the value placed on a QALY (ranging from £0 to £100 000, in £2000 increments) multiplied by the incremental QALY and then subtracting the incremental cost. For each of the 1000 resamples and each QALY value, the groups were ranked in terms of their net benefits, and these data then used to produce the CEACs.

Sensitivity analyses were conducted by (a) basing analyses on available data without multiple imputation using ordinary least-squares regression models (given different numbers with complete cost and QALY data); (b) changing the fixed element of the therapy cost (i.e. hardware, maintenance, etc.) from £50 per recipient of therapy to £75, £100, £125 and £150; and (c) increasing and decreasing therapist costs by 25 and 50% to reflect the possibility of staff of different grades delivering therapy or extra non-contact time being required.

## Results

Out of 642 people who were assessed, 345 eligible participants were randomised: 114 to the AV-EXT group, 116 to the AV-BRF group and 115 to TAU. Demographic and clinical characteristics at randomisation were similar for each group, both for the randomised sample and the complete case sample (Table 1). Follow-up service use and cost data were available for all participants at baseline. At 16-week follow-up these were available for 98 (84.5%) AV-BRF, 96 (84.2%) AV-EXT and 104 (90.4%) TAU participants. At 28-week follow-up the figures were 98 (84.5%) AV-BRF, 91 (79.8%) AV-EXT and 102 (88.7%) TAU. EQ-5D-5L baseline data were available for 115 (99.1%) AV-BRF, 112 (98.2%) AV-EXT and 115 (100%) TAU participants. At 16-week follow-up the figures were 96 (82.8%), AV-BRF, 92 (80.7%) AV-EXT and 99 (86.1%) TAU. At 28-week follow-up the figures were 94 (81.0%) AV-BRF, 83 (72.8%) AV-EXT and 99 (86.1%) TAU.

**Table 1:** Sample characteristics

Characteristics	TAU (*n* = 115)	AV-BRF (*n* = 116)	AV-EXT (*n* = 114)
Age (years); mean (s.d.)	38.7 (12.8)	39.4 (13.3)	40.8 (13.7)
Gender; *n* (%)			
Male	69 (60.0)	72 (62.1)	71 (62.3)
Female	44 (38.3)	43 (37.1)	42 (36.8)
Other	2 (1.7)	1 (0.9)	1 (0.9)
Ethnicity; *n* (%)			
White	69 (60.0)	63 (54.3)	71 (62.3)
Black	19 (16.5)	19 (16.4)	19 (16.7)
South Asian	12 (10.4)	9 (7.8)	6 (5.3)
Other	15 (13.0)	25 (21.6)	18 (15.8)
Marital status; *n* (%)			
Single	86 (74.8)	87 (75.0)	86 (75.4)
In a relationship, married or cohabiting	20 (17.4)	20 (17.3)	16 (14.0)
Widowed or divorced	9 (7.8)	9 (7.7)	12 (10.5)
Diagnosis; *n* (%)			
F20 – Schizophrenia	54 (47.0)	52 (44.8)	45 (39.5)
F22 – Persistent delusional disorders	2 (1.7)	1 (0.9)	0 (0)
F23 – Acute and transient psychotic disorders	2 (1.7)	0 (0)	2 (1.8)
F24 – Induced delusional disorder	0 (0)	1 (0.9)	0 (0)
F25 – Schizoaffective disorders	6 (5.2)	9 (7.8)	12 (10.5)
F28 – Other non-organic psychotic disorders	4 (3.5)	3 (2.6)	1 (0.9)
F29 – Unspecified non-organic psychosis	31 (27.0)	35 (30.2)	41 (36.0)
F31 – Bipolar affective disorder	3 (2.6)	1 (0.9)	4 (3.5)
F32.3 – Severe depressive episode with psychotic symptoms	11 (9.6)	14 (12.1)	8 (7.0)
Not available or not applicable	2 (1.7)	0 (0)	1 (0.9)
PSYRATS-AH-Distress; mean (s.d.)	15.7 (2.8)	15.7 (2.7)	15.9 (2.8)
PSYRATS-AH-Total; mean (s.d.)	30.6 (4.4)	30.1 (4.7)	30.1 (4.4)

TAU, treatment as usual; AV-BRF, brief form of AVATAR therapy; AV-EXT, extended form of AVATAR therapy; PSYRATS-AH, Psychotic Symptoms Rating Scale.

Tables 2 –4 show service use and costs (other than for mental health admissions and the intervention) for available cases at each time point. In the period prior to baseline, the most commonly used services were contacts with general practitioners (GPs), psychiatrists and mental health nurses (Table 2). Around a third of participants had contacts with both psychologists and early intervention teams. There were no substantial differences between the three groups in terms of numbers of participants using services. In the period between baseline and 16- and 28-week follow-ups, GPs, psychiatrists and mental health nurses continued to be used more than other services. Use of psychologists fell in each group, although it was slightly higher for TAU participants. The mean number of service contacts among participants with at least one contact at baseline and each follow-up indicated that intensity of service use did not differ noticeably between groups (Table 3). The highest non-in-patient and non-therapy costs at each time point were observed for psychiatrists (Table 4). Most other costs were relatively low, given the small numbers using these services (most participants had zero costs for most services). However, the cost of GP contacts was low even though upwards of half of the participants used this service.

**Table 2:** Number (%) of participants using services (excluding AVATAR therapy) at each time point

Service	Baseline			16-week follow-up			28-week follow-up		
	TAU (*n* = 115)	AV-BRF (*n* = 116)	AV-EXT (*n* = 114)	TAU (*n* = 104)	AV-BRF (*n* = 98)	AV-EXT (*n* = 96)	TAU (*n* = 102)	AV-BRF (*n* = 98)	AV-EXT (*n* = 91)
General practitioner	66 (57)	58 (50)	69 (60)	53 (51)	47 (48)	59 (61)	51 (50)	42 (43)	54 (59)
Psychiatrist	77 (67)	85 (73)	69 (60)	66 (63)	70 (71)	59 (61)	51 (50)	58 (59)	49 (54)
Other doctor	22 (19)	22 (19)	24 (21)	14 (13)	25 (26)	17 (18)	18 (18)	17 (17)	9 (10)
Psychologist	34 (30)	32 (28)	30 (26)	21 (20)	14 (14)	10 (10)	20 (20)	14 (14)	9 (10)
Drug/alcohol advisor	8 (7)	9 (8)	13 (11)	4 (4)	4 (4)	12 (13)	6 (6)	6 (6)	10 (11)
Counsellor/therapist	9 (8)	9 (8)	6 (5)	8 (8)	5 (5)	5 (5)	5 (5)	4 (4)	2 (2)
Crisis team worker	26 (23)	21 (18)	33 (29)	18 (17)	15 (15)	25 (26)	11 (11)	11 (11)	15 (16)
EI team worker	39 (34)	38 (33)	43 (38)	33 (32)	25 (26)	33 (34)	31 (30)	27 (28)	27 (30)
Social worker	20 (17)	26 (22)	34 (30)	25 (24)	20 (20)	27 (28)	19 (19)	25 (26)	14 (15)
Mental health nurse	75 (65)	81 (70)	76 (67)	72 (69)	65 (66)	63 (66)	71 (70)	60 (61)	59 (65)
Occupational therapist	17 (15)	19 (16)	11 (10)	15 (14)	13 (13)	16 (17)	10 (10)	12 (12)	17 (19)
Community centre	15 (13)	13 (11)	12 (11)	7 (7)	10 (10)	16 (17)	7 (7)	15 (15)	17 (19)
Day centre/day hospital	3 (3)	2 (2)	6 (5)	6 (6)	4 (4)	8 (8)	6 (6)	6 (6)	3 (3)
Drop-in centre	2 (2)	3 (3)	3 (3)	3 (3)	2 (2)	0 (0)	0 (0)	3 (3)	3 (3)
Self-help/support group	13 (11)	15 (13)	16 (14)	13 (13)	10 (10)	8 (8)	7 (7)	12 (12)	12 (13)
Group activity	13 (11)	7 (6)	6 (5)	8 (8)	12 (12)	9 (9)	8 (8)	9 (9)	8 (9)
Adult education	6 (5)	5 (4)	5(4)	5 (5)	11 (11)	5 (5)	5 (5)	7 (7)	2 (2)
Other services	1 (1)	1 (1)	0	4 (4)	2 (2)	5 (5)	0 (0)	3 (3)	3 (3)
Non-psychiatric hospital admissions	2 (2)	4 (3)	3 (3)	4 (4)	4 (4)	3 (3)	1 (1)	4 (4)	2 (2)

TAU, treatment as usual; AV-BRF, brief form of AVATAR therapy; AV-EXT, extended form of AVATAR therapy; EI, early intervention.

**Table 3:** Mean (s.d.) number of service contacts for those using services at each time point

Service	Baseline			16-week follow-up			28-week follow-up		
	TAU (*n* = 115)	AV-BRF (*n* = 116)	AVT-EXT (*n* = 114)	TAU (*n* = 104)	AV-BRF (*n* = 98)	AV-EXT (*n* = 96)	TAU (*n* = 102)	AV-BRF (*n* = 98)	AV-EXT (*n* = 91)
General practitioner	2.8 (3.0)	3.3 (3.2)	2.9 (2.8)	2.6 (1.8)	2.6 (2.1)	2.3 (1.6)	2.1 (1.2)	3.0 (4.0)	2.4 (1.9)
Psychiatrist	2.8 (4.7)	1.8 (1.7)	2.4 (2.2)	1.9 (1.4)	2.0 (1.5)	2.4 (2.8)	1.8 (1.3)	1.8 (1.0)	2.0 (1.8)
Other doctor	2.5 (2.1)	2.0 (1.7)	2.0 (2.1)	1.6 (0.9)	1.7 (1.0)	2.2 (1.7)	1.4 (0.8)	2.1 (1.3)	2.0 (1.3)
Psychologist	5.0 (3.1)	4.6 (3.7)	4.9 (4.3)	5.4 (3.6)	3.6 (3.9)	2.8 (1.8)	4.6 (2.9)	4.2 (2.2)	1.3 (0.5)
Drug/alcohol advisor	4.4 (3.3)	8.8 (8.5)	2.9 (2.4)	5.8 (4.6)	5.8 (4.3)	4.0 (3.5)	4.0 (3.7)	2.3 (0.8)	3.6 (3.3)
Counsellor/therapist	4.7 (3.1)	5.9 (4.4)	4.7 (6.2)	4.7 (4.4)	5.6 (5.5)	6.0 (3.1)	2.8 (1.8)	3.5 (2.1)	2.0 (0.0)
Crisis team worker	10.8 (13.5)	10.5 (10.6)	7.5 (12.5)	10.1 (22.3)	6.1 (8.7)	8.3 (12.4)	11.2 (12.9)	5.0 (4.8)	7.3 (10.6)
EI team worker	9.1 (8.4)	6.8 (7.1)	7.1 (7.7)	7.8 (8.3)	6.6 (7.1)	7.1 (6.2)	6.9 (7.8)	5.1 (5.3)	7.9 (7.8)
Social worker	5.8 (3.8)	5.5 (3.9)	4.8 (3.5)	5.5 (5.2)	6.3 (4.3)	6.1 (5.5)	4.8 (4.1)	4.6 (4.6)	5.7 (4.6)
Mental health nurse	6.7 (6.7)	7.4 (13.5)	6.9 (7.2)	9.0 (15.3)	6.3 (5.6)	7.0 (8.5)	5.7 (6.6)	4.4(3.1)	5.5 (4.4)
Occupational therapist	4.3 (3.7)	6.0 (5.8)	2.5 (3.0)	4.7 (3.7)	6.8 (8.2)	4.9 (3.7)	5.9 (7.3)	6.3 (7.1)	3.8 (3.4)
Community centre	3.6 (3.1)	2.9 (3.0)	7.3 (10.1)	4.7 (3.5)	3.7 (3.6)	4.8 (4.0)	2.7 (2.5)	3.5 (2.3)	5.3 (5.5)
Day centre/day hospital	9.7 (14.2)	1.5 (0.7)	3.4 (2.3)	4.7 (7.6)	3.5 (3.0)	4.6 (5.4)	1.8 (1.0)	3.0 (2.5)	5.0 (6.1)
Drop-in centre	13.5 (17.7)	3.3 (2.5)	2.0 (1.0)	24.3 (31.4)	4.5 (3.5)	–	–	13.7 (19.4)	2.3 (1.5)
Self-help/support group	5.8 (7.1)	8.5 (12.5)	4.1 (3.5)	9.7 (7.7)	14.6 (15.3)	8.6 (4.4)	4.0 (4.3)	11.0 (14.6)	5.3 (4.2)
Group activity	11.6 (24.1)	13.1 (12.7)	22.2 (19.0)	13.8 (20.2)	17.8 (12.8)	33.3 (57.2)	7.6 (8.0)	19.0 (16.0)	8.8 (7.1)
Adult education	15.0 (16.6)	19.0 (18.2)	11.8 (15.8)	13.0 (7.1)	19.7 (17.1)	9.0 (9.2)	24.8 (15.9)	12.6 (8.7)	3.5 (0.7)
Other	1.0 (0)	25.0 (0)	–	3.8 (3.6)	3.0 (1.4)	6.4 (4.6)	–	5.7 (5.5)	2.3 (0.6)
Non-psychiatric hospital admissions	2.0 (0)	4.0 (4.2)	7.0 (9.5)	1.3 (0.5)	9.3 (9.9)	12.7 (15.3)	6.0 (0)	4.8 (6.8)	1.0 (0)

TAU, treatment as usual; AV-BRF, brief form of AVATAR therapy; AV-EXT, extended form of AVATAR therapy; EI, early intervention.

Means and standard deviations apply only to those with at least one contact.

**Table 4:** Mean service costs (pounds sterling) at each time point (complete case sample)

Service	Baseline			16-week follow-up			28-week follow-up		
	TAU (*n* = 115)	AV-BRF (*n* = 116)	AVT-EXT (*n* = 114)	TAU (*n* = 104)	AV-BRF (*n* = 98)	AV-EXT (*n* = 96)	TAU (*n* = 102)	AV-BRF (*n* = 98)	AV-EXT (*n* = 91)
General practitioner	78 (137)	82 (207)	75 (116)	81 (160)	55 (98)	64 (82)	50 (87)	74 (167)	79 (216)
Psychiatrist	598 (1207)	424 (718)	448 (657)	394 (537)	411 (662)	435 (717)	290 (485)	303 (443)	288 (465)
Other doctor	92 (242)	109 (348)	86 (233)	36 (110)	82 (175)	78 (253)	50 (137)	82 (264)	45 (218)
Psychologist	157 (322)	122 (303)	148 (387)	128 (315)	48 (195)	34 (138)	102 (253)	66 (189)	13 (51)
Drug/alcohol advisor	11 (52)	40 (260)	21 (95)	12 (87)	8 (57)	27 (127)	12 (70)	6 (26)	28 (148)
Counsellor/therapist	22 (93)	24 (114)	17 (125)	23 (117)	22 (129)	20 (98)	10 (53)	11 (60)	2 (16)
Crisis team worker	44 (140)	27 (88)	30 (85)	36 (207)	21 (88)	28 (93)	24 (133)	8 (37)	18 (61)
EI team worker	118 (481)	90 (323)	83 (187)	71 (176)	37 (118)	75 (169)	101 (469)	52 (151)	78 (196)
Social worker	29 (91)	38 (101)	39 (109)	40 (140)	34 (85)	43 (113)	22 (72)	35 (106)	24 (80)
Mental health nurse	94 (171)	104 (256)	85 (133)	117 (201)	88 (146)	80 (148)	81 (138)	56 (90)	62 (112)
Occupational therapist	127 (534)	148 (550)	45 (214)	124 (442)	168 (782)	158 (481)	57 (301)	122 (501)	110 (359)
Community centre	3 (11)	3 (21)	5 (29)	3 (13)	4 (19)	8 (33)	1 (8)	4 (16)	23 (160)
Day centre/day hospital	6 (58)	1 (7)	5 (24)	4 (25)	3 (19)	5 (31)	2 (9)	1 (6)	3 (30)
Drop-in centre	6 (58)	3 (27)	<1 (3)	17 (145)	1 (9)	0 (0)	0 (0)	7 (66)	1 (7)
Self-help/support group	13 (67)	20 (112)	10 (29)	26 (103)	29 (126)	12 (57)	7 (35)	23 (108)	16 (62)
Group activity	29 (208)	11 (56)	13 (66)	20 (113)	30 (107)	26 (125)	6 (31)	26 (116)	9 (41)
Adult education	38 (243)	29 (193)	2 (10)	25 (142)	92 (363)	8 (47)	54 (291)	46 (229)	3 (18)
Other	<1 (1)	5 (49)	0 (0)	2 (12)	1 (10)	4 (22)	0 (0)	<1 (2)	1 (5)
Non-psychiatric hospital admissions	20 (154)	81 (589)	108 (995)	28 (150)	222 (1489)	232 (1840)	35 (350)	114 (898)	13 (86)

TAU, treatment as usual; AV-BRF, brief form of AVATAR therapy; AV-EXT, extended form of AVATAR therapy; EI, early intervention.

The AV-BRF group had a mean (s.d.) 5.1 (2.4) sessions of therapy, and the AV-EXT had a mean (s.d.) of 8.2 (4.4) sessions. The mean (s.d.) costs of therapist time were £420 (£198) for AV-BRF and £674 (£365) for AV-EXT. Over the combined follow-up period, mental health in-patient admissions occurred for a few participants (8 AV-BRF, 7 AV-EXT and 9 Tau). For those who were admitted, the mean number of days during the follow-up period was 70.0 for AV-BRF, 27.9 for AV-EXT and 76.7 for TAU. Mean costs for this in-patient use were substantially less for AV-EXT (£583) than for either AV-BRF (£1646) or TAU (£2046).

Total costs at baseline were very similar between groups (Table 5). Across all participants with cost data, mean follow-up costs were £4271 for TAU, £4624 for AV-BRF and £4070 for AV-EXT. Following multiple imputation, the incremental costs compared with TAU, controlling for baseline costs and site, were £319 for AV-BRF (95% CI, –£1558 to £2496) and –£196 for AV-EXT (95% CI, –£1912 to £1519). The incremental costs for those with available cost data were £435 for AV-BRF (95% CI, –£1770 to £3319) and £208 for AV-EXT (95% CI, –£1583 to £1929).

**Table 5:** Cost-effectiveness results

Parameters	TAU	AV-BRF	AV-EXT
Mean (s.d.) baseline total costs (£)	2695 (5267)	3213 (7582)	2695 (5300)
Mean (s.d.) psychiatric in-patient costs (£)	2046 (10 369)	1646 (8007)	583 (2617)
Mean (s.d.) AVATAR therapy costs (£)	0 (0)	677 (314)	1065 (557)
Mean (s.d.) total follow-up costs (£)	4271 (11 164)	4624 (8473)	4070 (4060)
Mean (s.d.) QALYs over follow-up	0.3089 (0.1439)	0.3341 (0.1678)	0.3287 (0.1377)
Missing data analysis (SUR)			
Incremental costs compared with TAU (£)^a^	–	319 (95% CI, −1558 to 296)	−196 (95% CI, −1912 to 1519)
Incremental QALYs compared with TAU	–	0.0159 (95% CI, −0.0103 to 0.0422)	0.0173 (95% CI, −0.0049 to 0.0395)
Incremental cost-effectiveness ratio compared with TAU (£)	–	20 016	Dominates
Available case analysis (OLS)			
Incremental costs compared with TAU (£)^a^	–	435 (95% CI, −1770 to 3319)	208 (95% CI, −1583 to 1929)
Incremental QALYs compared with TAU^b^	–	0.0171 (95% CI, −0.0089 to 0.0416)	0.0165 (95% CI, −0.0077 to 0.0389)
Incremental cost-effectiveness ratio compared with TAU (£)	–	25 439	12 606

TAU, treatment as usual; AV-BRF, brief form of AVATAR therapy; AV-EXT, extended form of AVATAR therapy; QALYs, quality-adjusted life years; SUR, seemingly unrelated regression; OLS, ordinary least squares.

a. Adjusting for baseline costs and study site.

b. Adjusting for baseline 5-level version of the EuroQol 5-Dimension questionnaire score and study site.

### Primary health economic analyses

At baseline, the EQ-5D-5L quality of life scores were comparable across the three groups (Table 6). By the 16-week follow-up, both AVATAR groups showed an improvement in their scores. However, by the 28-week mark there was a slight decrease in scores for both AVATAR groups compared with the 16-week point. EQ-5D-5L scores for TAU remained relatively constant. Based on the multiple imputation analysis, over the combined follow-up, AV-BRF resulted in 0.0159 (95% CI, −0.0103 to 0.0422) more QALYs than TAU, and AV-EXT in 0.0173 (95% CI, −0.0049 to 0.0395) more QALYs than TAU (Table 5). The analysis based on available cases showed incremental QALY gains of 0.0171 (95% CI, −0.0089 to 0.0416) for AV-BRF and 0.0165 (95% CI, −0.0077 to 0.0389) for AV-EXT.

**Table 6:** Mean (s.d.) health-related quality of life scores derived from the 5-level version of the EuroQol 5-Dimension questionnaire

Time point	TAU	AV-BRF	AV-EXT
Baseline	0.5379 (0.2989)	0.5289 (0.3120)	0.5322 (0.2959)
16-week follow-up	0.5531 (0.2755)	0.5787 (0.3010)	0.5855 (0.2734)
28-week follow-up	0.5116 (0.3137)	0.5719 (0.3267)	0.5626 (0.2948)

TAU, treatment as usual; AV-BRF, brief form of AVATAR therapy; AV-EXT, extended form of AVATAR therapy.

**Fig. 1:**
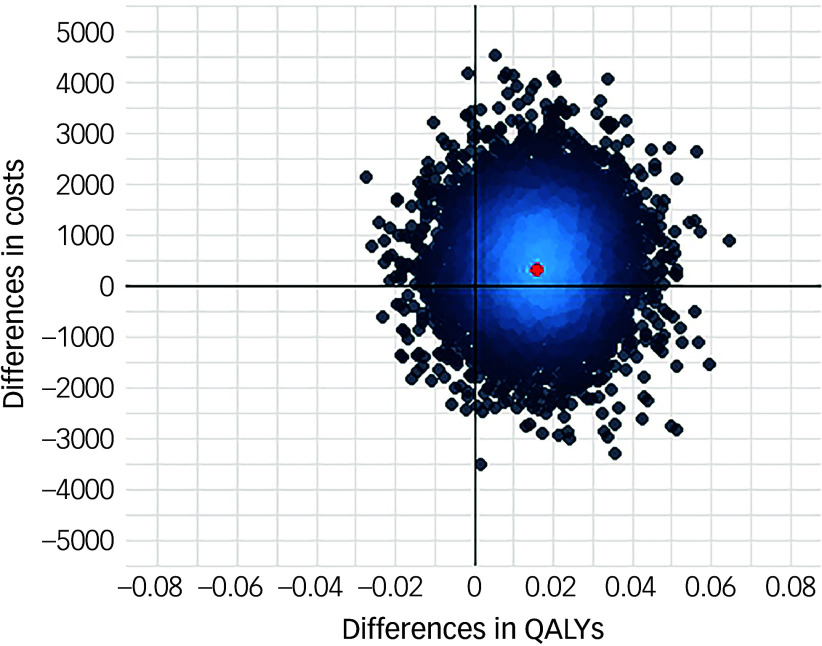
Cost-effectiveness plane, brief form of AVATAR therapy versus treatment as usual. QALYs, quality-adjusted life years.

**Fig. 2:**
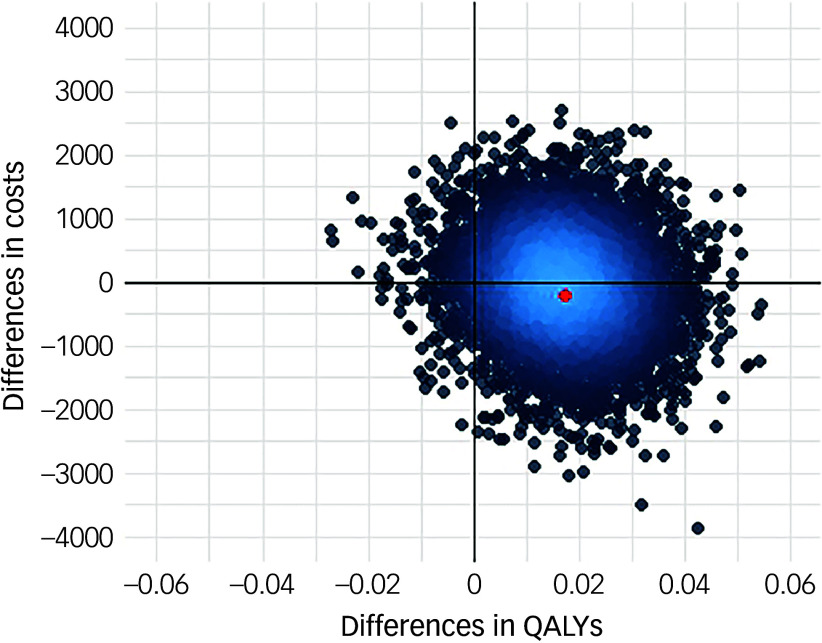
Cost-effectiveness plane, extended form of AVATAR therapy versus treatment as usual. QALYs, quality-adjusted life years.

**Fig. 3:**
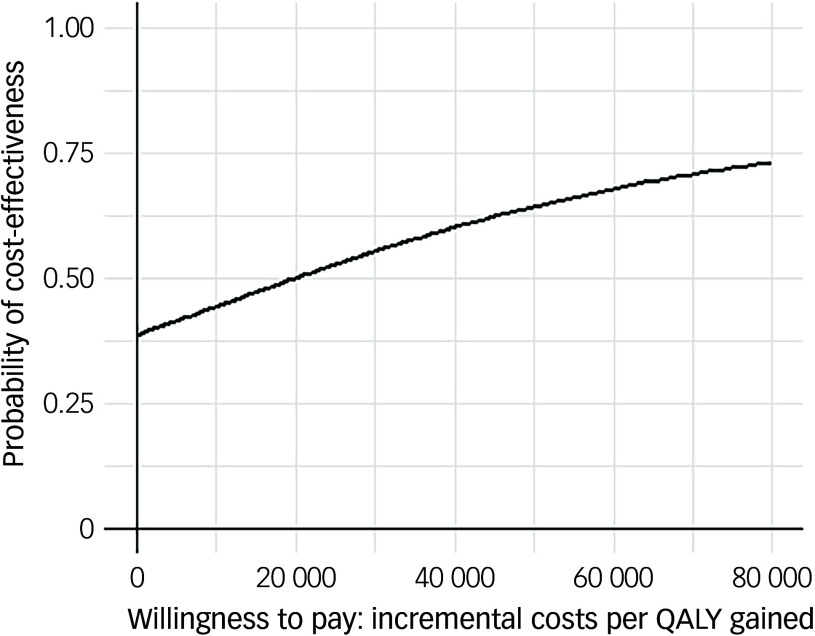
Cost-effectiveness acceptability curve (brief form of AVATAR therapy versus treatment as usual). QALYs, quality-adjusted life years.

**Fig. 4:**
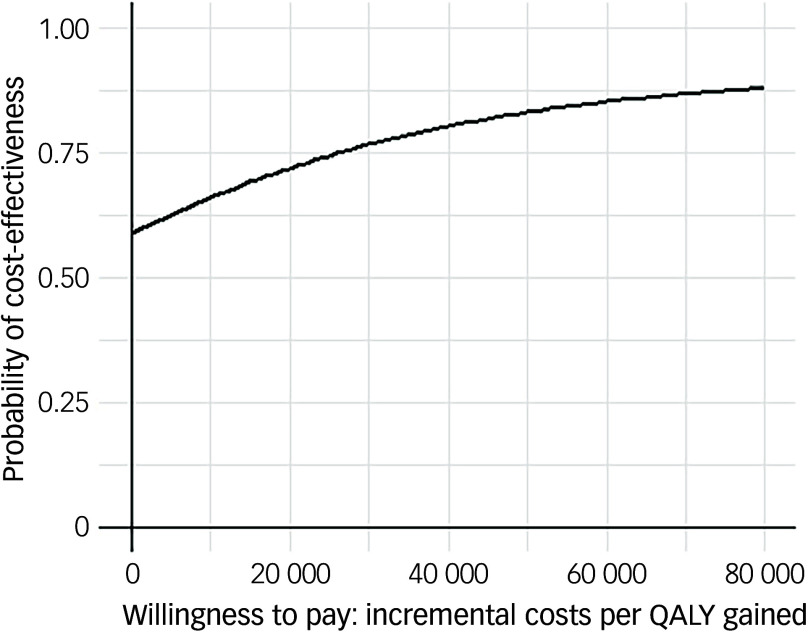
Cost-effectiveness acceptability curve (extended form of AVATAR therapy versus treatment as usual). QALYs, quality-adjusted life years.

Following multiple imputation for missing data, AV-BRF resulted in higher costs and more QALYs than TAU, with an ICER of £20 016 per QALY. AV-EXT dominated TAU (it had lower costs and produced more QALYs). Figures 1 and 2 show cost-effectiveness planes indicating uncertainty in the cost-effectiveness results. In Figure 2, for AV-EXT the most incremental cost–QALY combinations are shown at the top-right (higher costs and better outcomes) and bottom-right (lower cost and better outcomes) quadrants. A similar pattern emerged for AV-BRF, although the incremental costs were generally higher. The cost-effectiveness acceptability curves (Figs. 3 and 4) show that AV-EXT has a higher likelihood of being the most cost-effective option compared with TAU for all values placed on a QALY. AV-BRF has a greater likelihood of being the most cost-effective option compared with TAU for values placed on a QALY exceeding around £20 000.

At a threshold of £20 000 per QALY (the lower threshold used by the National Institute for Health and Care Excellence (NICE)), AV-BRF had a probability of 0.4943 of being more cost-effective than TAU, and this increased to 0.5534 for a threshold of £30 000. For AV-EXT the probabilities were 0.7188 and 0.7662, respectively.

### Sensitivity analyses

When the non-staff costs of therapy (equipment, software, maintenance, etc.) were decreased from £50 per participant to £25, the ICER for AV-BRF compared with TAU fell to £18 657. If these costs increased to £75–£150, the ICER for AV-BRF compared with TAU then increased to £21 374–£25 449. For these alternative non-therapist costs, AV-EXT always dominated TAU.

If therapist costs are reduced by 50%, the ICER for AV-BRF then becomes £52; if these are reduced by 25%, the ICER for AV-BRF then becomes £10 034; and if they are increased by 25 and 50% then the ICER is £29 997 and £39 979, respectively. AV-EXT dominates TAU regarding changes to therapy costs unless these are increased by 50%, when ICER becomes £18 173.

## Discussion

This study reports evidence on the cost utility of the brief and extended forms of AVATAR therapy, compared with TAU. We have shown that AV-EXT is cost-effective compared with TAU, in that it has lower costs and produces more QALYs. AV-BRF results in higher costs and has an ICER slightly above £30 000 (the upper threshold used by NICE in England and Wales). These findings offer supplementary evidence to the main trial outcomes, in which AV-EXT therapy exceeded the prespecified threshold for a clinically significant post-treatment change on the primary outcome (AV-BRF was just below), and demonstrated improvements in secondary outcomes prioritised by voice-hearers, including measures of well-being and recovery. However, it is important to note that while AV-EXT emerged as the more cost-effective option compared with TAU, the analysis is based on sample averages meaning that, for some, AV-BRF may be more cost-effective and indeed for others TAU may be more appropriate.

These results were almost entirely influenced by cost differences. Both forms of AVATAR therapy resulted in more QALYs than TAU, but these incremental gains were very small. Although AV-EXT is a more expensive form of therapy than AV-BRF, the AV-EXT trial arm had lower psychiatric in-patient costs during the follow-up period, which resulted in overall cost savings. The use of QALYs and EQ-5D-5L to generate these has long been a source of debate regarding mental health studies.^21,22^ The very small QALY gains for AV-EXT and AV-BRF compared with TAU mean that either health-related quality of life was largely unaffected by receipt of the interventions, or that EQ-5D-5L was not sensitive to change in this study. In support of the latter explanation, the lack of observed change in EQ-5DL stands in contrast to the pattern of relevant secondary outcomes reported in the trial, which included a significant positive impact on well-being. The main outcomes from this trial show that, at 16-week follow-up, AB-BRF and AV-EXT improved significantly on the Warwick–Edinburgh Mental Wellbeing Scale, by 2.19 and 6.83 points, respectively, compared with TAU.^12^ At 28 weeks, AV-EXT improved by 5.1 points compared with TAU while the improvement for AV-BRF (2.03) was not statistically significant. Self-reported recovery showed significant improvements for both groups compared with TAU at both time points. Given that the primary aim was to reduce distress caused by voices, it is perhaps expected to see the biggest impacts on the anxiety and depression domains of EQ-5D-5L. EQ-5D-5L continues to be used widely in mental health studies, although mental health-specific alternatives such as the Recovering Quality of Life questionnaire are also used.^23^

Although AV-EXT produced gains compared with TAU and appeared to be cost-effective, it is important to note that completion of treatment for this group was 57.9% compared with 81.9% for AV-BRF. This did not affect the results, in that we attached costs to actual therapy time; however, if completion rates were higher so would be the costs. Of course, it may also then be the case that outcomes could be improved further.

The current study was not designed to consider the complexities involved when moving beyond clinical research trials into real-world deployment of AVATAR therapy. Implementation is recognised as a challenge that many digital interventions, regardless of efficacy and cost-effectiveness evidence, fail to overcome.^24–26^ In this study, therapy was delivered by a relatively small number of therapists (19 therapists delivering therapy across 8 NHS trusts), while sustained roll-out of therapy will require training of more people.

AVATAR therapy has recently been recommended for NHS deployment to support the collection of real-world evidence.^27^ Therefore, the next steps will be to test the effectiveness and cost-effectiveness of AVATAR therapy when delivered in diverse routine care settings, and to understand the barriers to, and facilitators of, wider implementation.

### Limitations

Although this was a large trial and with a reasonable rate of follow-up, there are limitations in regard to the economic evaluation. First, to estimate care costs we partly relied on self-report data from participants. This was unavoidable given the lack of linked data covering all services that were relevant. However, the use of mental health services was checked against records. Second, while the study was large, the use of in-patient care may have been unbalanced due to the small numbers receiving this. Findings for AV-BRF may have been impacted by a small number of in-patient high-cost admissions exerting a disproportionate influence on service costs. This is frequently an issue with economic evaluations, where cost data are inevitably skewed. Third, the main outcome measure used in the analysis was QALYs derived from EQ-5D-5L (see discussion above).

In conclusion, this study has shown that providing AVATAR therapy in an extended form could be cost-effective compared with TAU. There is, however, much uncertainty in the results, and the finding in favour of the therapy is almost entirely due to the lower costs in that group. There is limited evidence currently to suggest that the brief form of AVATAR therapy is cost-effective, although the costs for AV-BRF may have been disproportionately influenced by a relatively small number of high-cost admissions. Based on the recent NICE-EVA recommendation and associated evidence generation plan, the next steps are to test the implementation of AVATAR therapy, including the provision of further evidence of effectiveness and cost-effectiveness, when delivered in diverse routine care settings. The evidence presented in this paper converges with main trial outcomes to suggest that future implementation is primarily guided by the AV-EXT protocol.

## Supporting information

McCrone et al. supplementary material McCrone et al. supplementary material

## Data Availability

Data will be made accessible following publication of this paper. A request can be made by academic or clinical researchers to research.data@kcl.ac.uk for the purpose of conducting non-commercial, ethically approved research. The research data team will review the request against the conditions set out in the data access agreement. An initial response to requests will be formulated within 1 month. A data access agreement will be drawn up before data can be shared.

## Supplementary material

The supplementary material is available online at https://doi.org/10.1192/bjo.2025.10925
