# Supplementary material for: Economic evaluation of digitally supported therapy for people with psychosis who hear distressing voices: the AVATAR2 trial
Source: BJPsych Open. 2025 Dec 15;12(1):e13. doi: 10.1192/bjo.2025.10925 (PMC12724094; doi:10.1192/bjo.2025.10925)
Supplement: McCrone et al. supplementary material [file S2056472425109253sup001.docx]

**Appendix. Unit costs.**

| **Service** | **Cost** | **Unit source** |
| --- | --- | --- |
| General Practitioner | £33 / contact | Unit Costs of Health and Social Care 2021/2 (p. 66) |
| Psychiatrist | £235/ contact | Unit Costs of Health and Social Care 2021/2 (p. 39) |
| Other Doctor | £235 / contact | Unit Costs of Health and Social Care 2021/2 (p. 39) |
| Clinical Psychologist | £75/hour | Unit Costs of Health and Social Care 2021/2 (p. 59) |
| Drug/Alcohol Advisor | £88 / hour | Unit Costs of Health and Social Care 2021/2 (p. 24) |
| Other counsellor / Therapist  (*cost of psychologist used) | £114/ contact | NHS reference cost 2021/2 |
| Home Treatment/ Crisis Team Worker | £43 / hour | Unit Costs of Health and Social Care 2021/2  Assumption: same price with the mental health nurse |
| Early Intervention Worker | £43 / hour | Unit Costs of Health and Social Care 2021/2  Assumption: same price with the mental health nurse |
| Social Worker | £50 / hour | Unit Costs of Health and Social Care 2021/2 (p. 77) |
| Mental Health Nurse | £43 / hour | Unit Costs of Health and Social Care 2021/2 |
| Occupational Therapist | £99/ contact | NHS reference cost 2021/2 |
| Community Centre | £14 / hour | Unit Costs of Health and Social Care 2021/2 (p. 18) |
| Day Centre/ Day Hospital | £14 / hour | Unit Costs of Health and Social Care 2021/2 (p. 18) |
| Drop-in Centre | £14 / hour | Unit Costs of Health and Social Care 2021/2 (p. 18) |
| Self-help / Support Group | £14 / hour | Unit Costs of Health and Social Care 2021/2 (p. 18) |
| Class Group / Leisure Centre | £14 / hour | Unit Costs of Health and Social Care 2021/2 (p. 18) |
| Impatient day for mental health reason | £341 / bed day | Unit Costs of Health and Social Care 2021/2(p.16) |
| Inpatient day for physical health reason | £586.59 / bed day | <https://www.ncbi.nlm.nih.gov/pmc/articles/PMC7045184/> |
